# Optimizing water use efficiency in greenhouse cucumber cultivation: A comparative study of intelligent irrigation systems

**DOI:** 10.1371/journal.pone.0311699

**Published:** 2024-10-21

**Authors:** Faeze Behzadipour, Mahmoud Ghasemi-Nejad-Raeini, Saman Abdanan Mehdizadeh, Morteza Taki, Bijan Khalili Moghadam, Mohammad Reza Zare Bavani

**Affiliations:** 1 Department of Agricultural Machinery and Mechanization, Agricultural Sciences and Natural Resources University of Khuzestan, Mollasani, Iran; 2 Department of Soil Sciences, Faculty of Agricultural Sciences, Agricultural Sciences and Natural Resources University of Khuzestan, Mollasani, Iran; 3 Department of Horticultural Sciences, Faculty of Agricultural Sciences, Agricultural Sciences and Natural Resources University of Khuzestan, Mollasani, Iran; Ardakan University, ISLAMIC REPUBLIC OF IRAN

## Abstract

This study investigated the use of an intelligent irrigation system for greenhouse cucumber cultivation, aiming to manage water consumption efficiently. During the initial phase, irrigation was tested at four levels: 80%, 90%, and 100% of Field Capacity (FC), and Conventional Flood Irrigation (CFI). Data on environmental conditions and water usage were meticulously recorded. Optimal yields and crop quality (measured by size and firmness) were achieved at CFI and 100% FC, with CFI consuming the most water (0.148 m^3^/m^2^) Consequently, 100% FC was identified as the best practice, informing the intelligent system’s calibration in the subsequent phase. This adjustment resulted in reduced water consumption and a 15.6% improvement in Water Use Efficiency (WUE) over CFI. Additionally, by examining the product performance and the color characteristics, chlorophyll content, and photosynthesis of the leaves, it was observed that the quality and optimal water supply and the product performance were maintained in the smart irrigation system. The study concludes that, considering long-term outcomes, the intelligent irrigation system is preferable to CFI, offering significant water savings and enhanced WUE without compromising crop quality.

## 1. Introduction

Competition between water resources has intensified due to increasing population and further economic development, changes in weather, and irregular rainfall, so irrigation water supply is facing serious crises in the agricultural field [1]. The global population is estimated to be around 9 to 10 billion by 2050, and therefore water demand will increase [2]. Sustainable use of water resources is a major challenge to ensuring food security for present and future generations because agriculture is the largest sector of water consumption [3]. So, increasing water productivity is essential for sustainable agricultural development through technologies that produce more food per drop of water [4]. Farmers can be helped to cope with drought in arid and semi-arid regions by optimizing crop yield and water productivity [5] as well as through integrated irrigation management [6].

Some studies assessed the water use in agriculture such as soil mulching [7], maximizing irrigation efficiency by using drip irrigation [8], the interactions of under-irrigation and compost, and changes in temperature and plant breeding [9]. On the other hand, managing methods in the consumption of agricultural inputs (especially water) and its improvement, as well as methods to increase productivity, are among the challenges faced in recent years in this area. Finally, this issue requires a correct and deep understanding of existing biological stresses and the plant’s response to them [10] Methods to increase water saving and sustainability in agriculture include Early diagnosis of water stress and implementing appropriate timing methods for irrigation which must be done before reduced performance and occurrence of irreparable damage [11]. Also, greenhouse cultivation is common all over the world. Greenhouse cultivation is common all over the world (due to global warming and unstable water conditions) and provides suitable conditions (especially environmental conditions) for the production of various products, such as products with high prices or products that are grown in a controlled and closed structure in the off-season [11–13] so yields of crops in greenhouses are reported about 2 to 3 times higher than their yields in fields (open farms) [14].

Water is a fundamental input in agricultural production and holds a crucial role in the sector’s development. Given the limitation of land and water resources, and the significant consumption of water in agriculture, it is imperative approaches to enhance efficiency and reduce water usage, alongside the expansion of greenhouse cultivation [15, 16]. The cucumber, scientifically known as *Cucumis sativus L*,. belongs to the gourd family (Cucurbitaceae). It is one of the oldest vegetables globally and and ranks as the most important and costly vegetable after tomatoes and cabbage in many countries [17]. As the plant with the largest area under greenhouse cultivation. cucumbers exhibit high water consumption and are cultivated both in summer and winter. In Iran, the cultivated area of greenhouse crops exceeds 12,000 hectares, with vegetables and summer crops constituting over 75% of this area. Among these, accounts for more than 70% of the vegetable and summer crop, making greenhouses the primary cultivation method. In this study, an intelligent irrigation system was developed and implemented for greenhouse cucumber cultivation. This system assessed and measured water consumption, productivity and the quantitative characteristics of cucumbers in two stages. Parameters such as environmental, soil, and plant water and humidity levels, as well as water consumption, were measured to evaluate the system’s effectiveness.

### 1.1. Precision irrigation

Precise irrigation as a critical concept in the field of agriculture improves the efficiency of water consumption and as a result, stabilizes or increases crop yield, which has an important role in agriculture especially in precision agriculture [18]. The main goal of accurate and intelligent irrigation systems is to provide the amount of water required by plants and agricultural products [19] according to the temperature and humidity conditions of the soil, moisture and performance characteristics of plant [20]. The cucumber is one of the warm-season plants that this crop is sensitive to soil water availability. Therefore, methods of proper irrigation and planning are important for greenhouse cucumber cultivation to have high yields of product and improve water use efficiency [12]. In China, under drip irrigation, maximum yields for cucumber crops were obtained when the potential of the soil matrix was above -20 kPa in open space or the content of soil water was above 85% of field capacity [21].

### 1.2. Intelligent irrigation system

Researchers showed that water consumption was reduced by 70–80% by installing an intelligent irrigation system equipped with IoT-connected humidity and temperature sensors in green tea greenhouse cultivation [22]. Also, other researchers performed optimal mathematical modeling for a precision field green pepper irrigation system and compared it with an irrigation system equipped with sensors and a predetermined soil moisture content threshold [23, 24]. They reported that the amount of water consumption is 18,370 and 15,622 liters, respectively, in the two systems of precision irrigation and the system equipped with sensors. The amount of water savings was 14.9% in the system equipped with the sensor [25]. Some studies introduced a system that works based on IoT. This system can control the water consumed by measuring the humidity of the environment, the temperature of the air, and the moisture of the soil [26].

Based on the results, the plant’s water requirement was stated to be between 600 and 800 mm per day, although the temperature range needs to be clarified as the stated range of 40 to 60°C is not plausible for plant life. In another research, the amount of water stored (per day) was 14.590 ml for 90 Lilium flowers by using an intelligent irrigation system [27]. In a separate study, the use of a wireless sensor network was explored to reduce the amount of water used for irrigating corn crops. This system received the relevant information from the sensors of temperature, humidity, wind, and light, then according to the water needs of the crop, issued irrigation commands that were sent for the axes (pipes) that those are according to the requirements water for the product. The system performance was reported at about 85% (that was along with the irrigation system of pipe). In a study, the consumption of water was reported to be 11% in uncontrolled irrigation lower than in automatic irrigation. but a higher percentage of optimization can be done due to the irrigation with automatic control in this system and can improve the determination of areas that need more irrigation [28].

Artificial Neural Networks (ANNs) are increasingly being utilized in water use irrigation systems to enhance efficiency and sustainability [29]. By analyzing vast amounts of data [30], such as soil moisture levels, weather forecasts and crop requirements, ANNs can predict the optimal amount of water needed for irrigation [31]. This predictive capability helps in minimizing water wastage and ensuring crops receive adequate hydration [32]. Additionally, ANNs can adapt to changing environmental conditions, making real-time adjustments to irrigation schedules [33]. This technology not only conserves water but also boosts crop yields and reduces operational costs.

### 1.3. Water use efficiency

A research, compared drip irrigation in cucumber crops to furrow irrigation in greenhouses and reported that drip irrigation in greenhouses reduced irrigation water by almost 50% and increased cucumber yield and economic benefits by approximately 3 to 4% compared with furrow irrigation [12]. A related research investigated the effect of water stress at 40, 60, and 80 c-bar on growth indices and yield of greenhouse cucumber [34]. The results showed that the highest yield was 37 and 39 kg/m^2^ in 40 and 60 c-bar treatments, respectively, and the lowest yield was related to 80 c-bar suction treatment with 20 kg.m^-2^ due to higher moisture stress. Also, soil moisture stress did not affect the morphological characteristics of cucumber (length and diameter) despite reducing water consumption. In another study, drip irrigation and different amounts of water (three levels of 60, 80, and 100% of the total water required by the plant) on performance, WUE and quality characteristics of tomatoes was investigated [35]. The results showed that the highest yield was obtained 62.265 tons per hectare in the treatment with irrigation level, 100% of the water required by the plant. Also, the highest water use efficiency was obtained at 7.88 kg.m^-3^ from the treatment of 80% of the water supply. In a study, the effect of water shortage on yield and water use efficiency of cucumber and tomato greenhouse crops in three treatments of full irrigation, 80% and 90% of full irrigation was evaluated [36]. According to the results, the maximum yield of cucumbers and tomatoes was 144.6 and 197.3 tons per hectare under full irrigation and the yield of both crops decreased with low irrigation in both treatments (80 and 90%). Also, the highest water use efficiency is 53.2 and 55.5 kg.m^-3^ in the treatment with full irrigation for cucumber and tomato crops respectively, and the lowest water use efficiency showed a decrease of 31% and 20% respectively for cucumber and tomato in the treatment of 80%. In a related research, the effect of drip irrigation in comparison with conventional furrow irrigation on irrigation water volume, WUE and crop yield in greenhouse cucumber crops was evaluated [12]. According to the results, the average water use efficiency was 0.41 in conventional furrow irrigation but increased to 0.79 in drip irrigation. A research investigated WUE and crop yield by applying three irrigation treatments (80%, 100%, and 120% water requirement of the plant) in the growth period of the cucumber crop which the highest yield and consumption efficiency of water were reported in 120 and 80% summer treatments at 21.14 kg.m^-2^ and 49 kg.m^-3^, respectively [37]. Also, the volume of water consumed was obtained in the treatment of 80, 100, and 120% of the water requirement in winter 0.271, 0.335, and 0.408, and in summer 0.360, 0.43,9, and 0.524 m^3.^m^-2^ respectively. The effect of irrigation level was not significant on greenhouse cucumber yield in winter and summer cultivation and they reported that instead of full irrigation, 80% of the water requirement could be used.

### 1.4. Quantitative specifications of the product

In a study, the growth characteristics of greenhouse cucumbers under irrigation drought stress was investigated [38]. According to the reported results, the application of drought stress and increasing the irrigation cycle (from 3 to 5 days) greatly reduced the fresh and dry weight of fruit, diameter, and length of fruit, so that fruit wasn’t formed in the plant during the irrigation cycle every 7 days. Fruit tissue firmness, length, dry weight, and fresh weight of fruit had a decreasing trend (p<0.05) during the irrigation cycle every 5 days compared to the irrigation cycle every 3 days. Fruit diameters did not differ significantly (p>0.05) at 3 and 5-day irrigation cycles. Also, crop yield decreased with increasing irrigation cycle, so that yield at irrigation cycles of 3 and 5 days was reported at 5.95 and 5.16 kg per bush, respectively. In another study, the effect of superabsorbent on yield and water uses efficiency of greenhouse cucumbers under low irrigation condition was investigated [39]. The results indicated that low irrigation had a significant effect on all characteristics including bush height, chlorophyll content, length, diameter, and weight of fruit, root length, weight, yield, and water use efficiency. The highest fruit yield (3818.1 g. bush^-1^) was for fully irrigated plants and 0.4% superabsorbent. The highest water use efficiency (14.95 kg.m^-3^) was in the plant under low irrigation (6.2 mm height of irrigation water) and 0.4% superabsorbent. The maximum length and diameter of fruit were 16.69 cm and 28.2 mm, respectively in full irrigation treatment, and the lowest fruit length and diameter were 14.69 cm and 22.2 mm, respectively, in low irrigation treatment (6.2 mm of irrigation water).

## 2. Materials and methods

This research was carried out in a greenhouse with an area of 35m^2^ (7×5 meters) at the University of Agricultural Sciences and Natural Resources in northeastern Khuzestan, Iran from September 2021 to August 2023 (two stages of cultivation). The acknowledgment that the dataset was gathered by the article’s first author is crucial. The data acquisition has been done at the greenhouse in the mentioned university. This statement ensures that proper credit is given for the data collection effort. Furthermore, it should be noted that the dataset comprises solely observational data of the plants, with no experimental procedures performed.

The crop studied in this study was cucumber, and 4 irrigation levels were applied based on soil field capacity. The agricultural capacity of the soil was measured in the laboratory and the treatments include: treatment 1–80% of the agricultural capacity, treatment 2–90% of the agricultural capacity and treatment 3–100% of the agricultural capacity (equivalent to the agricultural capacity of the soil) and treatment 4- conventional flood irrigation) were selected.

Initially, a system was designed and set up using a variety of sensors (soil temperature and humidity, air temperature and humidity, plant temperature and light), pumps, flow meters, etc., so that it can accurately measure soil moisture and temperature, humidity and temperature of the ambient, plant temperature, amount of light and water consumption (Refer to section 2.1). At the same time, during the growing period of cucumber, all quantitative characteristics related to the product were collected and recorded. Also, some characteristics were measured including the color characteristics of the leaves and the amount of photosynthesis and chlorophyll.

The relevant analyses were performed and the system of irrigation was adjusted based on the optimal model obtained from the data recorded by the sensors at the best irrigation level. Irrigation was done in the second stage intelligently and the user did not interfere with irrigation. All characteristics of the cucumber crop were measured and analyzed in the second stage of cultivation and compared with the control (conventional flood irrigation in the second stage of cultivation). Statistical studies were performed and presented with SPSS and Excel software.

### 2.1. Design of intelligent irrigation system

In this research, sensors of capacitive humidity were used by using the properties of dielectric of water that measured the soil volumetric water. The analog channel collected the humidity values by the microcontroller. In addition, some sensors were used in the sensors set which included the non-contact infrared thermometer module, soil temperature, plant leaf temperature sensors, and the light sensor (**Table 1**). Two types of sensors (moisture and temperature sensors) were assembled at a depth of 10 cm (plant root area) and plant temperature sensors were assembled next to the leaves of the plant to collect their data. Other sensors were installed at an elevation of one meter from the ground including light, humidity and temperature sensors [40–42].

**Table 1:** Analysis of variance of irrigation on some cucumber crop features.

Source of variation	F value				
	Yield(kg)	Length (cm)	Diameter (mm)	firmness(kg)	Dry matter (%)
irrigation	6.014*	10.375*	48.711*	59.60*	32.249*

*, **, n.s are significant at the error level of 5%, 1%, and non-significance

**Fig 1:**
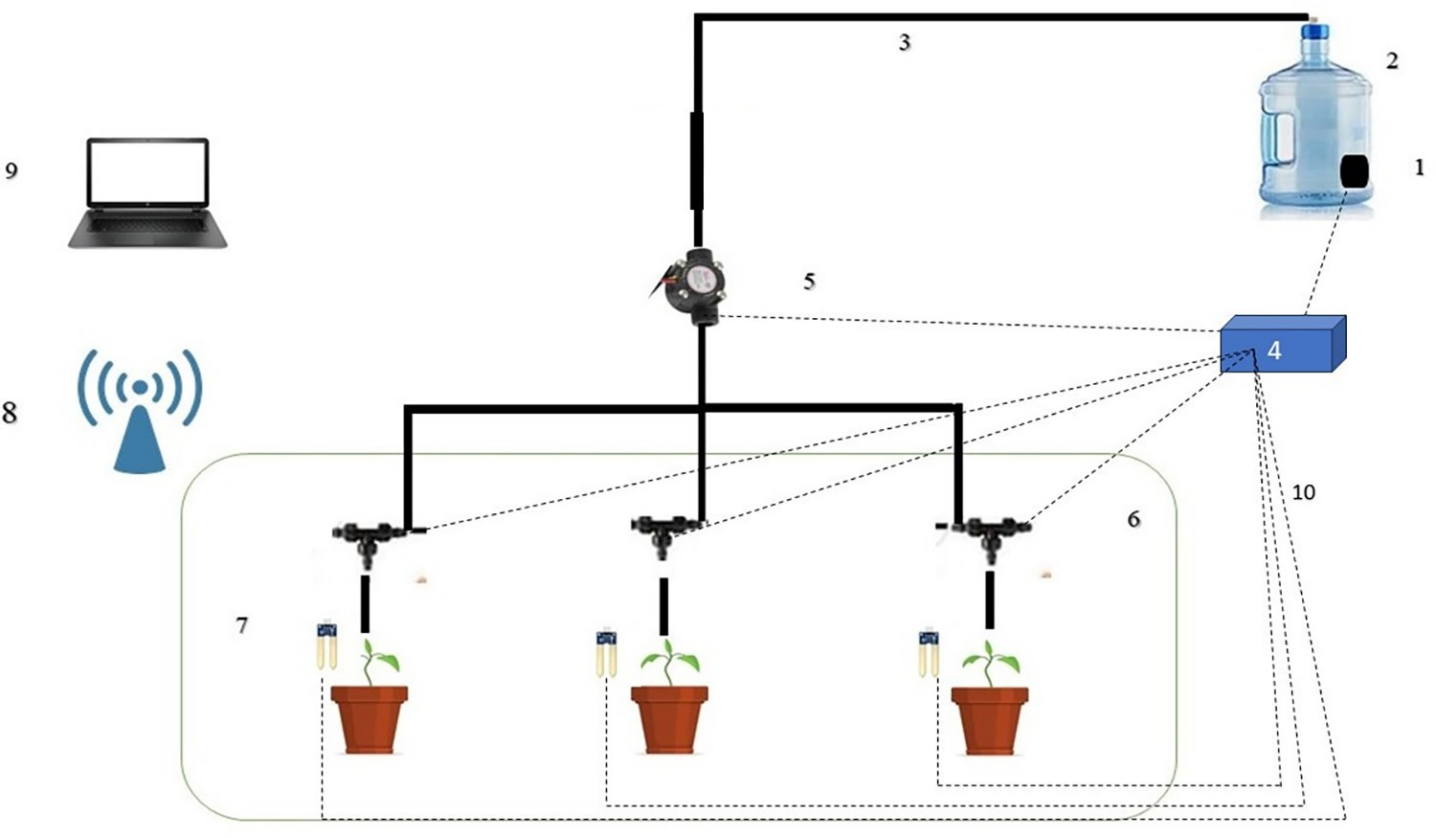
Schematic of intelligent irrigation system in the greenhouse. (1) Submersible pump (2) Water source (3) Pipe (4) Intelligent control box (5) Flow meter (6) Solenoid valve (7) Soil humidity and temperature sensor (8) Wi-Fi (9) Laptop, (10) wire.

**Fig 2:**
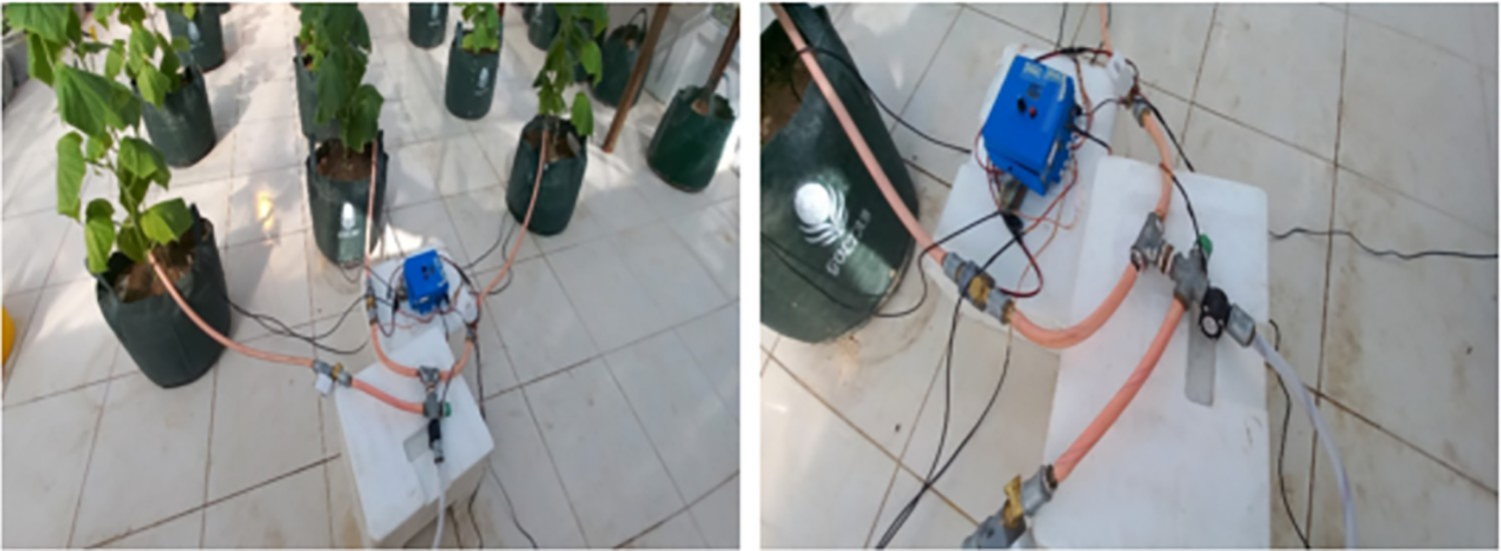
Pictures of the intelligent irrigation system in the greenhouse.

A rotary sensor was utilized to measure water consumption. All data was recorded by the sensors and stored in the device’s memory. After programming the microcontroller based on the optimal model, In the second stage of cultivation, the system was equipped with 3 solenoid valves and a submersible pump (AD180-1220D) which irrigation was done automatically (**Figs 1 and 2).** The parts used in the intelligent irrigation system included; sensors of capacitive humidity (Made in China, Model SEN0193; 0.1% accuracy), the non-contact infrared for the thermometer module (Made in China, Model MLX90615; 0.02 accuracy), soil temperature (Made in Taiwan, Model DS18B22; 0.1 degrees Celsius), plant leaf temperature sensors (Made in China, Model MLX90614; 0.1 accuracy), light sensor (Made in China, Model MAX44009; 0.01 accuracy), A rotary sensor (Made in China, Model YF-S201, 0.1 ml accuracy) and solenoid valves (Made in China, 12V, 3 pieces).

### 2.2. Crop features

To evaluate the quantitative characteristics of the crop, some parameters were measured [43] such as the percentage of fruit dry matter by drying the fruit in the oven and estimating the difference between fresh and dried fruit weight, fruit texture firmness, fruit length (with a ruler), fruit diameter (digital caliper) And crop performance. Fruit texture firmness was done with the texture analyzer (model of stable Microsystem- Gomalming- Surry- TA. XT Plus- UK, Prob: 6 mm, test speed: 1.7 mm s^-1^). Fruit yield was determined by harvesting at regular intervals, counting and weighing the samples [44–46].

### 2.3. Characteristics of plant leaves

Given the significant correlation between photosynthetic indices, leaf gas exchange, and the plant’s water status, these parameters were measured using a photosensitometer (model CI-340, LI-COR, Lincoln, Neb). The device quantified the raw photosynthesis rate of the plant in μmole/m^2^/s. To assess the chlorophyll index and the photosynthesis rate of plant leaves, two devices were employed: a chlorophyll meter (model SPED CL-01) and a photosynthesizer (model CI-340, LI-COR, Lincoln, Neb). Weekly sampling involved two leaves per plant. The photosynthesizer recorded the plant’s photosynthetic indices and gas exchanges. Measurements were conducted over two cultivation stages spanning two years. Additionally, to monitor the color changes in the plant leaves, photographs o was taken weekly. The collected data were statistically analyzed with SPSS 26.0.0.1 software and the results were transferred to Excel 2017 for graphing [45].

### 2.4. The water use efficiency

Water use efficiency (WUE) is the ratio of crop yield to the amount of irrigation water applied, which will be calculated by [47]:

$$Water\;\mathrm{use}\;\mathrm{efficiency}=\frac{Yield}{Total\;\mathrm{water}\;\mathrm{applied}}$$ (1)

In this equation, WUE: water use efficiency (kg.m^-3^), Yield: crop yield (kg. ha^-1^), total water applied: (m^3^.ha^-1^).

### 2.5. Intelligence irrigation system

To build an intelligent irrigation system, by using the data recorded from the sensors including (soil temperature and humidity, air temperature and humidity, and plant temperature) to predict soil moisture and apply irrigation, the best-selected model with predictive genetic programming was selected. The microcontroller of the irrigation system was adjusted according to the selected model. Therefore, based on the model provided to the system and the data received from the sensors, the plant’s water needs were detected and irrigated through electric valves. To evaluate the models predicted by genetic programming and check the error rate, the Root Mean Square Error (RMSE) and coefficient of determination (R^2^) were calculated [48, 49]:

$$R^{2}=\frac{{\left(\sum_{i=1}^{N}\left(y_{i}-{\tilde{y}}_{i})({\tilde{y}}_{i}-{\hat{y}}_{i}\right)\right)}^{2}}{\sum_{i=1}^{N}{\left(y_{i}-{\tilde{y}}_{i}\right)}^{2}\sum_{i=1}^{N}{\left({\tilde{y}}_{i}-{\hat{y}}_{i}\right)}^{2}}$$ (2)

$$RMSE=\sqrt{\frac{1}{N}\sum_{i=1}^{N}{\left(y_{i}-\tilde{y_{i}}\right)}^{2}}$$ (3)

## 3. Results and discussion

### 3.1. The first stage of cultivation

**Table 1** shows the results of variance analysis at the surface of 95% confidence related to the effect of irrigation treatments on some quantitative characteristics of the cucumber crop including yield (kg.m^-2^), fruit length (cm), fruit diameter (mm), texture firmness (kg) and dry matter of the fruit (%). Also, the Tukey test (HSD) was used to compare the mean of the measured characteristics at different levels of irrigation.

According to **Table 1,** irrigation treatments were significant at a 95% confidence level on crop yield, fruit length and diameter, tissue firmness, and percentage of fruit dry matter. According to results, the effect of different irrigation treatments was investigated on the average quantitative characteristics of the cucumber crop. Crop yield had a significant difference in treatments with irrigation of 80 and 90% of field capacity with irrigation treatments in the field capacity and the conventional flood irrigation.

**Fig 3:**
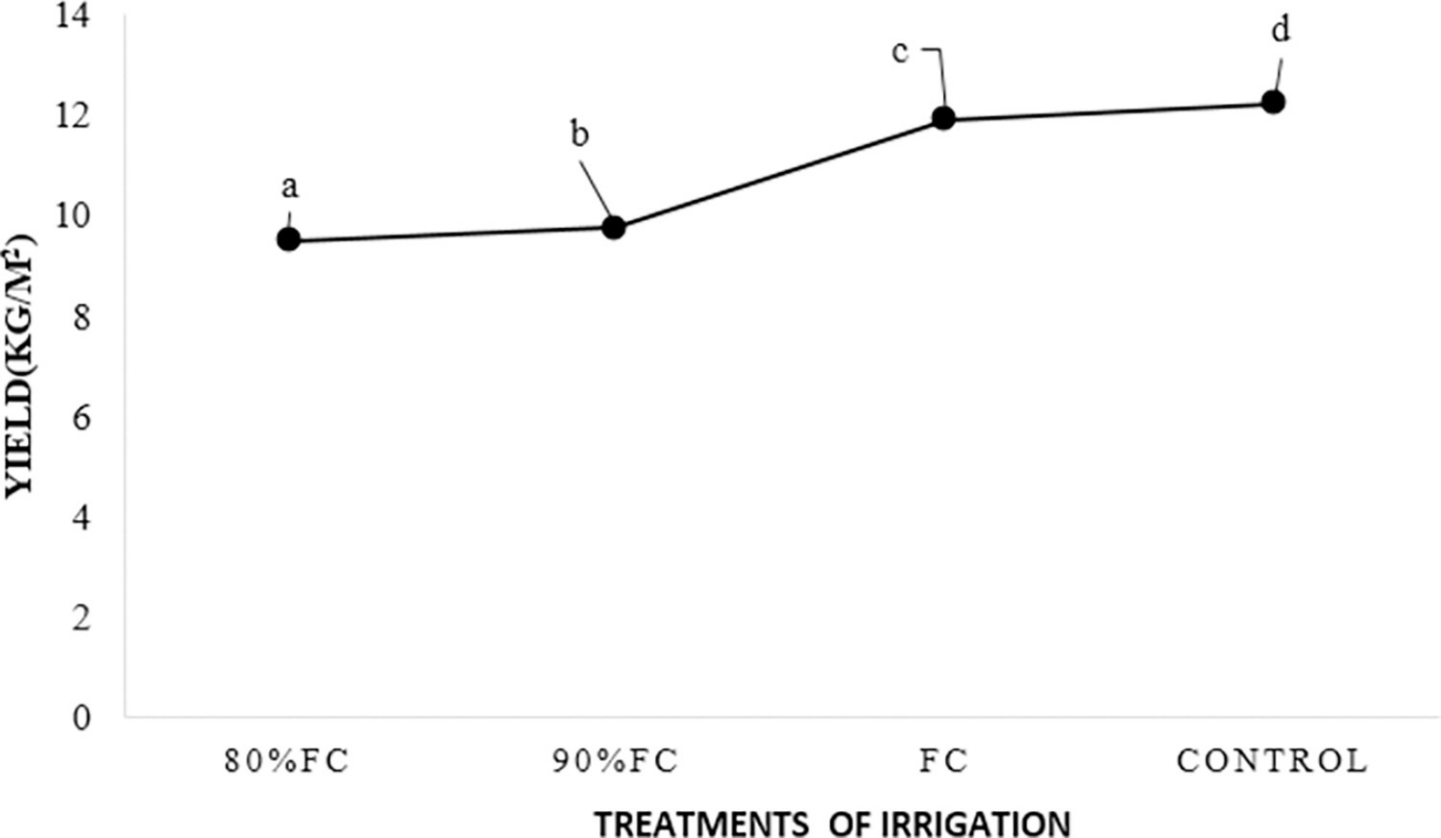
The effect of different irrigation treatments on crop yield (kg.m^-2^).

Based on the results of **Fig 3**, the highest and lowest yields were related to conventional flood irrigation and irrigation at 80% FC equal to 12.23 and 9.5 kg.m^-2^, respectively. Although there was a significant difference between conventional flood irrigation and irrigation treatment at the FC level, the yield of irrigation treatment at the FC level was equal to 11.89 kg.m^-2^ and there was no significant difference with conventional flood irrigation treatment.

**Fig 4:**
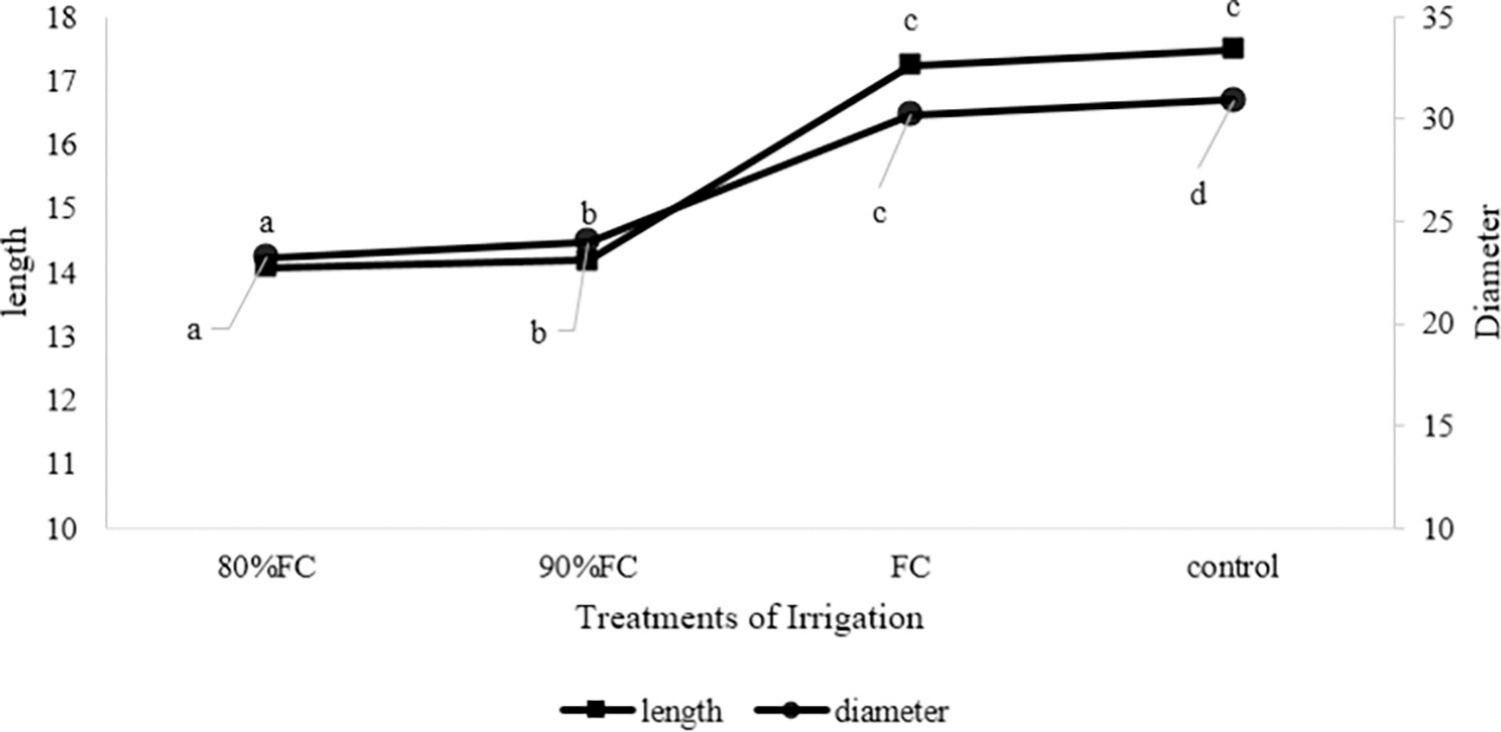
The effect of different irrigation treatments on the length (cm) and diameter (mm) of the crop.

Similarly, in **Fig 4** related to the length and diameter of cucumber crops, there is a significant difference between irrigation treatments of 80 and 90% of FC with irrigation treatments at the level of FC and conventional flood irrigation.

The average of the maximum and the minimum crop lengths was related to conventional flood irrigation and irrigation at 80% of field capacity of 17.467 and 14.07 cm. However, no significant difference was observed between treatments with conventional flood irrigation and field capacity level. Also, according to **Fig 3**, the maximum and minimum crop diameters were related to conventional flood irrigation and irrigation at 80% of field capacity equal to 30.90 and 23.24 mm respectively. Irrigation treatment at the field capacity level was slightly different from conventional flood irrigation treatment (30.187 mm).

**Fig 5:**
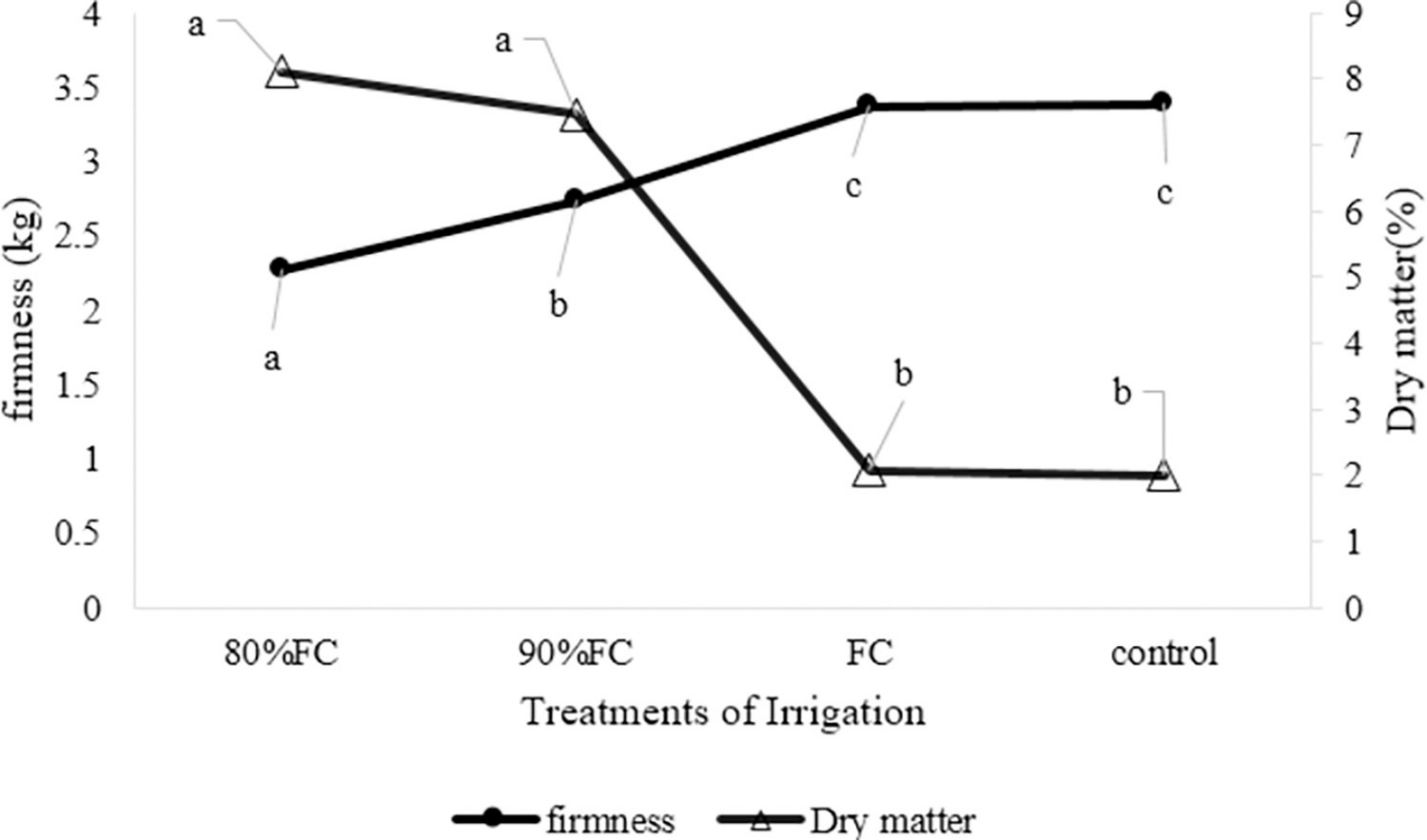
The effect of different irrigation treatments on tissue firmness (kg) and dry matter percentage of the crop.

**Fig 5** shows the effect of different irrigation treatments on tissue firmness and dry matter percentage of the cucumber crop. Irrigation treatment with 80% FC had the lowest amount of tissue firmness and treatments with irrigation at FC level and conventional flood irrigation had the highest tissue firmness with 2.27, 3.37, and 3.38 (kg), respectively. The percentage of dry matter in cucumber crops had the lowest amount for 80% and 90% FC treatments, 91.8%, and 92.5%, respectively, and had the highest amount of dry matter related to 100% FC and conventional flood irrigation (control) equal to 2.08% and 2.005%, respectively. As can be seen, the amount of irrigation had a significant effect on tissue firmness and dry matter of the crop, and if irrigation stress occurs for the plant, such as treatment of 80 and 90% FC, has a direct effect on the texture and dry matter of the crop. Fruit firmness and dry matter content improve in the absence of stress (such as FC and the conventional flood of treatments).

Water scarcity and drought stress affect plant performance at different stages of growth (including physiological activities of the plant) and reduce their speed. Decreased photosynthesis in leaves is another effect of reduced plant water availability and drought stress that occurred due to the relative reduction of stomatal protective cell inflammation and subsequent reduction in carbon dioxide uptake. All these factors prevent optimal growth in the plant and reduce the yield and quality of fruit production in the plant [38]. Stress and lack of irrigation reduced yield and growth of quantitative crop traits in the plant Similarly in the research of [12, 34–39].

**Fig 6:**
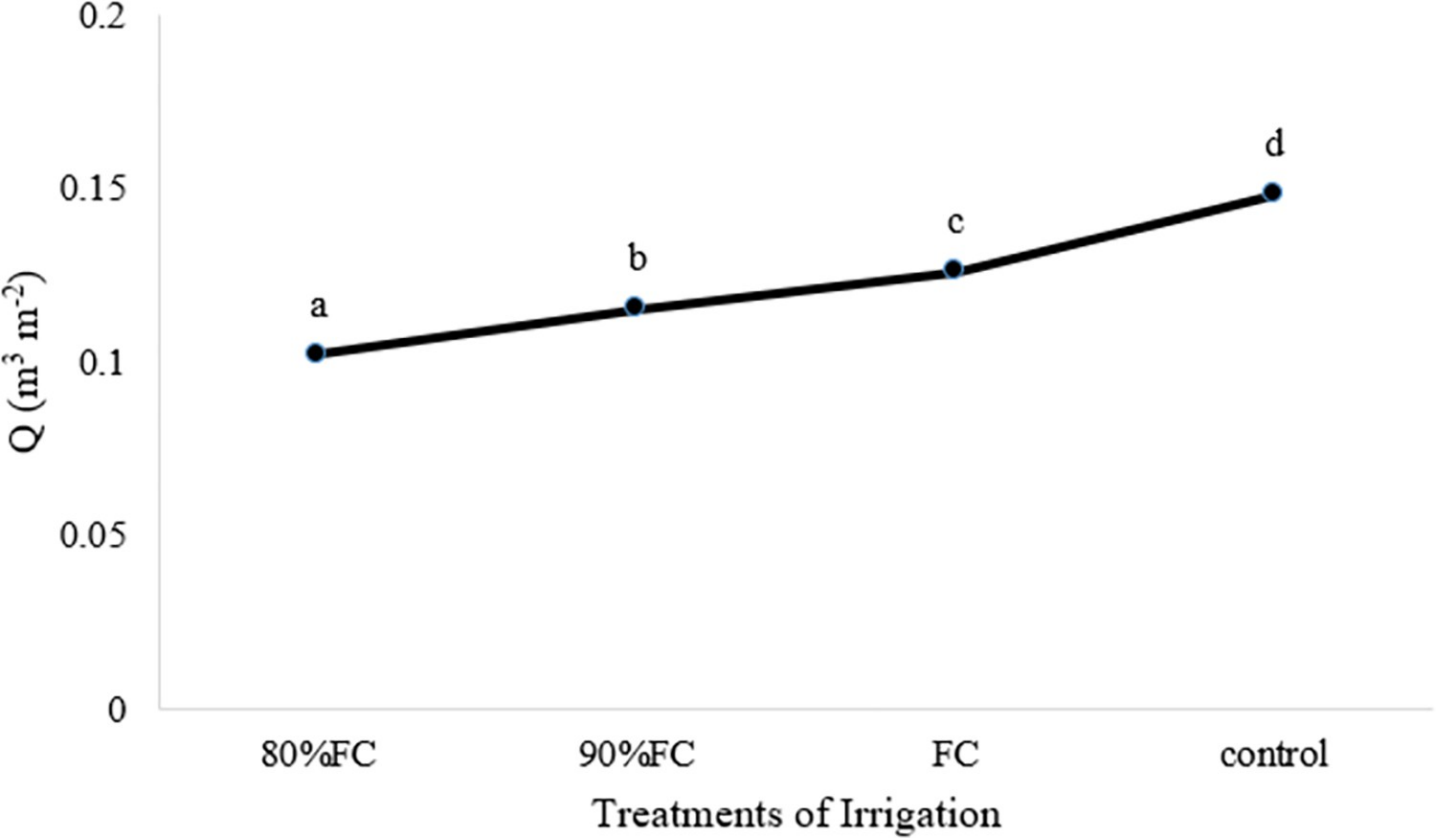
Amount of water consumption (m^3^m^-2^) in different treatments.

**Fig 6** shows the average water consumption per square meter. The lowest water consumption is related to irrigation treatment at the level of 80% FC (0.102 m^3^.m^-2^) and the highest amount of water consumption is related to conventional flood irrigation treatment (0.148 m^3^.m^-2^).

Therefore, conventional flood irrigation treatment (control) had a higher yield but had more water consumption than irrigation treatment at the field capacity level. On the other hand, the FC treatment was no different from the control treatment in features of texture firmness, length, and diameter of the cucumber. Therefore, FC irrigation treatment was selected as the optimal treatment and the system of irrigation was planned and adjusted based on the measured characteristics in the first year (moisture and temperature of soil, environment humidity and temperature, plant temperature, and light).

### 3.2. The second stage of cultivation

**Table 2** shows the models predicted by genetic programming. The best model was selected using genetic programming, which had a higher R^2^ (0.87) and a lower RMSE (0.19) was selected. The best-selected model with the highest coefficient of explanation (0.87) and the lowest error rate (0.19) was selected, which is under formula 6 (row 6) in **Table 2**.

**Table 2:** Prediction of soil moisture model (as output) in genetic programming with sensor data.

	predicted formula	R^2^	MSE	RMSE
1	6.18095×((X_6_)—(X_5_))-0.082796×(X_7_) + 33.445071	0.6482	1.5029	2.2587
2	-0.030255×((((X_6_) -((X_4_) × (X_5_))))) +14.618074	0.6304	0.9351	0.8744
3	-1.417192×((X_6_) + (X_4_)) +129.063305	0.7884	0.5171	0.2674
4	-1.114342× (X_6_) -1.476731 × (X_4_) -0.390532 × (X_5_) +	0.8064	0.5056	0.2557
	120.290384			
5	-1.219003 × (X_6_) -1.610459 × (X_4_) + 121.741966	0.8347	0.4869	0.2371
6	-0.911131 × (X_5_) +	0.8751	0.4438	0.1970
	-3.095896 × (X_4_) +			
	-0.192688 × (((((((X_4_)-[(X_6_)+(X_5_)])((([(X_6_)+(X_6_)]+[(X_6_)-(X_6_)])+([(X_5_)+(X_5_)]/(X_6_)))-(X_5_)))-(((([(x_6_)/(x_4_)]-[(x_6_)(x_4_)])((x_4_)/((x_4_)/(x_5_))))(([(x_5_)/(x_4_)](x_5_))/(x_6_)))+(x_4_)))-(x_5_))/(((([(x_4_)/(x_5_)][(x_6_)-(x_5_)])-(x_5_))/(x_5_))(([(x_5_)-(x_6_)](x_6_))+((x_4_)((([(x_6_)+(x_6_)][(x_4_)-(x_5_)])-([(x_6_)(x_6_)]/(x_5_)))/((x_5_)/(((x_5_)(x_6_))/((x_5_)+(x_5_)))))))))×(x_4_)) +			
	98.074742			

X_4_ = temperature of soil, X_5_ = temperature of ambient, X_6_ = humidity, X_7_ = plant temperature

Some parameters were measured and compared with conventional flood irrigation (control) by t-test at a 95% confidence level in the second period of cultivation, such as water consumption, crop yield, tissue firmness, diameter, and length of the crop (**Table 3**).

**Table 3:** Comparison of average by t-test between quantitative characteristics of cucumber and water consumption during the two stages of cultivation between intelligence irrigation and conventional flood irrigation (control).

Source of variation	t-Test value					
	Q	Yield	Length	Diameter	Dry weight	Firmness
Irrigation at F.C.	-25.779*	-1.429 ^n.s^	-1.095 ^ns^	-1.317 ^n.s^	-0.23 ^n.s^	0.209^n.s^
Standard Error	0.0005	0.02	0.1	0.05	0.3	0.05

*, **, ns are significant at the error level of 5%, 1% and non-significance

According to the results presented in **Table 3**, the amount of water consumed in the intelligent irrigation system had a significant difference in 95% confidence level with conventional flood irrigation (control). Also, product yield and diameter showed a significant difference at a 95% confidence level, but texture firmness, dry matter percentage, and product length did not differ significantly.

**Fig 7:**
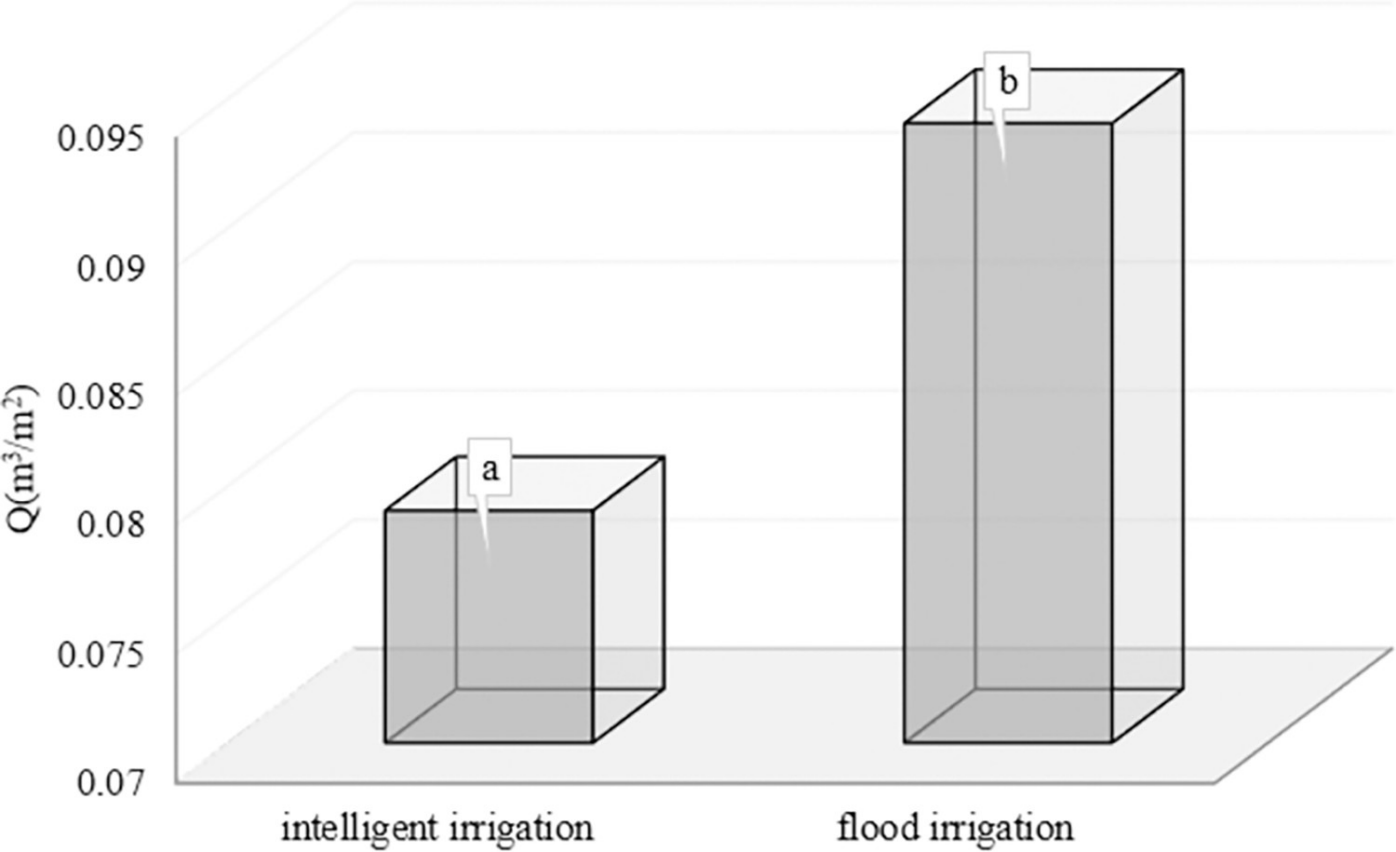
Water consumption (m^3^m^-2^) in the intelligent irrigation system and conventional flood irrigation (control).

On the other hand, the amount of water consumed in conventional flood irrigation (with 0.094 m^3^.m^-2^ is equivalent to 94 liters per m^-2^) is 16.2% more than irrigation with the intelligent system (with 0.079 m^3^.m^-2^ is equivalent to 79 liters per square meter) (**Fig 7**). Similarly, in studies by [12, 27, 28, 36], the consumption of water amount was Reduced and saved in intelligent and modern irrigation systems compared to conventional irrigation.

#### 3.2.1. Water use efficiency

There was a significant difference with the T-test at a 95% confidence level between water use efficiency in the intelligent irrigation system and conventional flood irrigation (control) (**Table 4**).

**Table 4:** Average water use efficiency in test treatments.

Source of variation	Average of parameters	
	Intelligent irrigation	Conventional flood irrigation
Water use efficiency (kgm^-2^)	76.27^a^	64.31^b^

**Fig 8:**
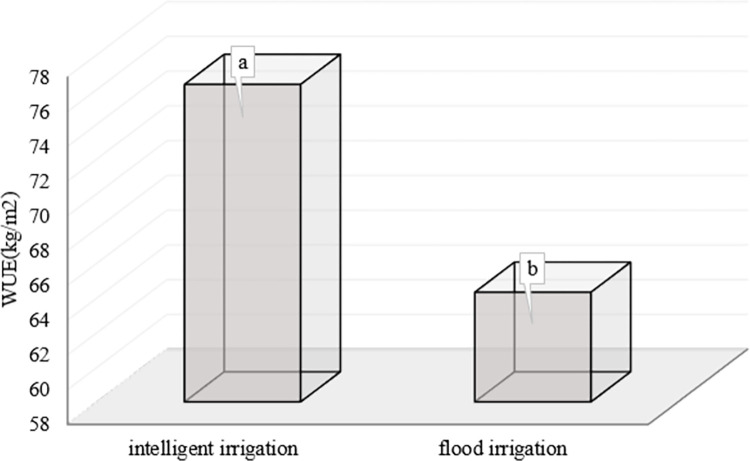
Water use efficiency in the second stage of cultivation between intelligence irrigation treatment with conventional flood irrigation treatment (control).

Irrigation not only reduces water consumption at the field capacity level but also has a higher water use efficiency than the control treatment as shown in **Fig 8**.

According to the results, the intelligent irrigation system increased the efficiency of water consumption by 15.6% compared to conventional flood irrigation. Therefore, in the discussion of water consumption efficiency, the intelligent irrigation system not only reduced water consumption but also improved water consumption efficiency and finally, can have the best results in economic issues for the farmer considering the importance of water consumption in today’s agriculture. similar results were reported in terms of improving water use efficiency in studies by [12, 35, 36].

#### 3.2.2. Investigating plant color indicators

T-test was performed to investigate the difference between rgb color indices in two irrigation treatments with intelligent system and conventional flood irrigation (control). According to the results presented in **Table 5**, no statistically significant difference was observed between the color indices in the two irrigation treatments and the quality of color in the leaves was at the same level.

**Table 5:** Statistical investigation of the effect of different irrigation treatments on the color characteristics r, g and b of plant leaves.

Source of variation	t-Test value			
		r indicat	g indicat	b indicat
Treatments of Irrigation		-0.423*	-0.317 ^n.s^	-0.509 ^ns^
Standard Error		0.008	0.016	0.016

*, **, ns are significant at the error level of 5%, 1% and non-significance

#### 3.2.3. Chlorophyll index and leaf photosynthesis rate

**Fig 9:**
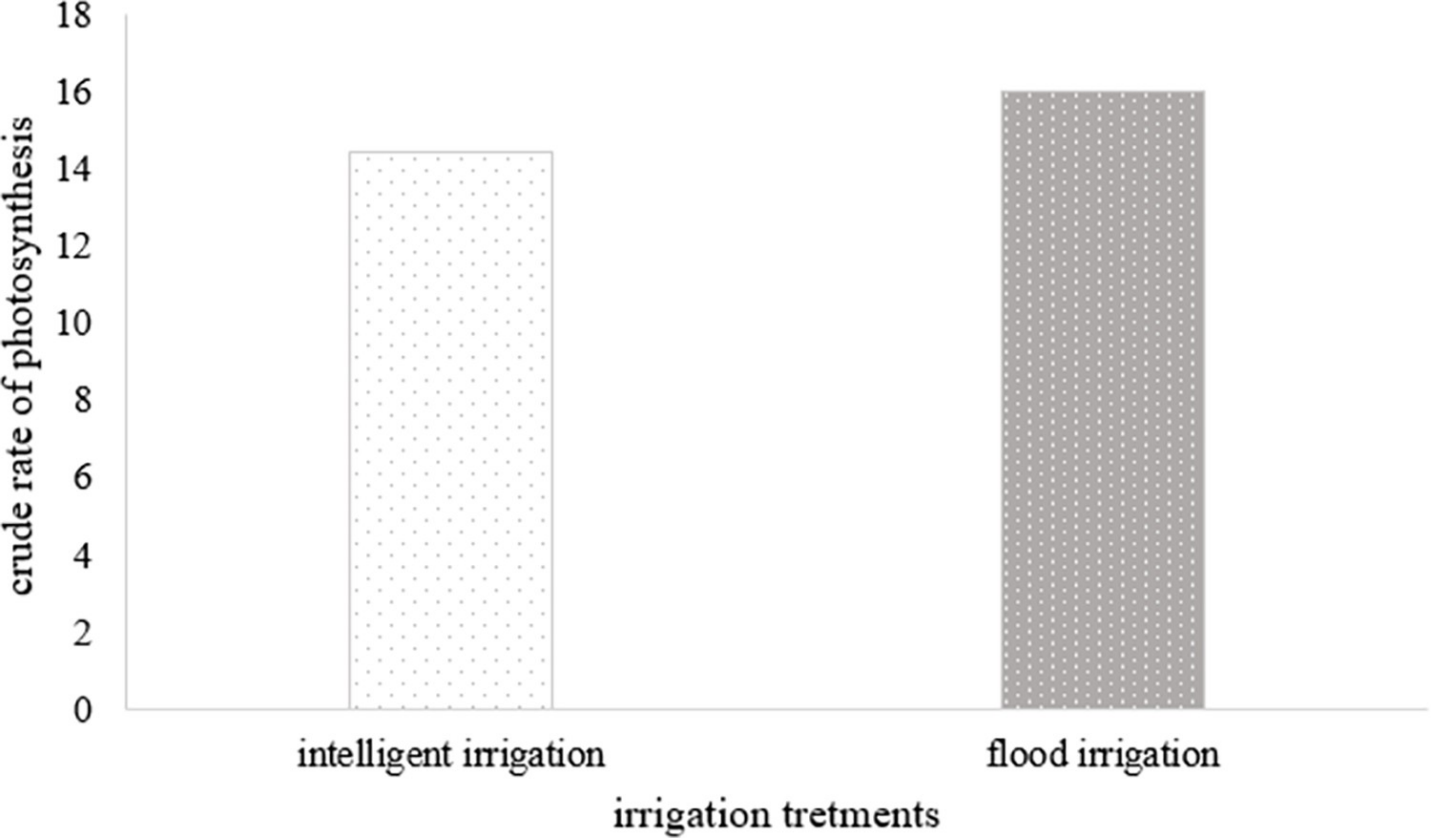
The raw rate of photosynthesis in plant leaves during the months of cultivation (second cultivation period) with intelligent irrigation system and conventional flood irrigation.

A statistical analysis was conducted to compare the chlorophyll index and photosynthesis rate between two treatments: conventional flood irrigation (control) and irrigation using intelligent system (Table 6). The results indicated a significant difference in the crude rate of photosynthesis between the two irrigation methods at the 5% error level (Fig 9), with values of 14.43 and 15.97 for the intelligent system and conventional flooding, respectively. However, no significant difference was observed in the chlorophyll index between the two irrigation methods at the 95% confidence level. This suggests that the leaves maintained their greenness and exhibited better photosynthesis while ensuring sufficient soil moisture for the plant.

**Table 6:** Statistical investigation of the effect of different irrigation treatments on the chlorophyll index and photosynthesis rate in plant leaves.

Source of variation	t-Test value	
		chlorophyll index	The crude rate of photosynthesis
Treatments of Irrigation		-0.37*	-7.55**
Standard Error		1.5	0.2

*, **, ns are significant at the error level of 5%, 1% and non-significance

**Fig 10:**
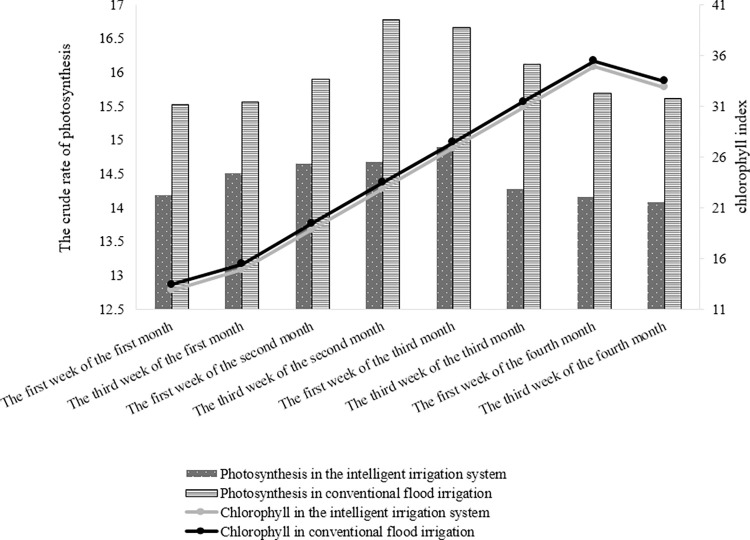
Changes in the chlorophyll index and raw photosynthetic rate of plant leaves in the two methods of conventional flood irrigation and irrigation with an intelligent system during the months of cultivation.

The comparison between the chlorophyll index and the crude rate of photosynthesis in two irrigation methods is shown in **Fig 10**. As it can be seen from the pictures, the upward trend of chlorophyll and photosynthesis decreased in the last months of cultivation and gradually these two indicators have experienced a downward trend, which can be caused by the weakening of the plant after several harvesting periods, as well as the tendency of the leaves to turn yellow and the plant to grow old. According to the results, the plant was not affected by any kind of dehydration, therefore, no difference was observed in the chlorophyll index of the leaves. [38, 50, 51] reported that drought stress and lack of irrigation had a significant effect on the leaf chlorophyll index and caused it to decrease, but according to the results presented in this research, due to lack of A decrease in the amount of irrigation and the absence of stress, no change in the chlorophyll index was observed.

## 4. Conclusion

Proper use of water resources and saving water consumption are essential, especially in arid and semi-arid regions. Since greenhouse cultivation increases water use efficiency and increases the quality and quantity of the product per unit of water, by increasing evaporation, increasing ambient humidity, and reducing temperature changes, in this study, favorable results were obtained from the use of a system of intelligent Irrigation for the cultivation of cucumber in the greenhouse. Irrigation with modern systems, including the intelligent irrigation system presented in this research, not only controls and reduces the amount of irrigation water consumption per square meter (16.2% increase in water consumption in conventional flood irrigation compared to intelligent irrigation) and increases by 15.6% the efficiency of water consumption in crop cultivation but also is profitable for farmers about the economic outlook. Intelligent system-controlled irrigation not only did not significantly change the functional but also reduced water consumption costs and controlled time, reduced labor costs for irrigation (wages and insurance), and has economic benefits for the farmer because irrigation takes place without user intervention. Therefore, this system is preferred and its use is recommended in the cultivation of greenhouse crops.

## Supporting information

S1 Data (XLSX)
